# Quantitative CT-derived small pulmonary vessel and erector spinae muscle parameters associated with acute exacerbation in COPD patients from a cold region: a dual-center study

**DOI:** 10.3389/fmed.2026.1931061

**Published:** 2026-09-29

**Authors:** Beibei Hou, Yiwen An, Peng Chen, Guokun Wang, Jinling Zhang

**Affiliations:** 1 Department of CT, The Second Affiliated Hospital of Harbin Medical University, Harbin, Heilongjiang, China; 2 Department of Radiology, The Fourth Hospital of Harbin, Heilongjiang, China

**Keywords:** acute exacerbation, chronic obstructive pulmonary disease, cold region, erector spinae muscle, nomogram, pulmonary small vessels, quantitative computed tomography

## Abstract

**Introduction:**

To investigate the association of quantitative CT-derived erector spinae muscle and small pulmonary vessel parameters with acute exacerbation of chronic obstructive pulmonary disease (AECOPD) in a cold-region population and construct a combined model to evaluate their joint discriminative value.

**Methods:**

This retrospective dual-center study included 273 patients with COPD from two centers in a cold region of northern China and divided them into non-AECOPD and AECOPD groups. Clinical characteristics, pulmonary function, and quantitative CT parameters, including erector spinae muscle cross-sectional area (CSA), volume, muscle attenuation, CSA/BMI, CSA × CT, and the percentage of small pulmonary vessel area <5 mm^2^ (%Vessel < 5), were collected. Factors independently associated with AECOPD were identified using logistic regression, and model performance was assessed by ROC analysis, nomogram, decision curve analysis, and calibration curve.

**Results:**

Compared with the non-AECOPD group, the AECOPD group had significantly lower CSA, volume, muscle attenuation, CSA/BMI, CSA × CT, and %Vessel < 5 (all *P* < 0.05). After adjustment for age, smoking history, pulmonary function, and center effect, %Vessel < 5 (OR = 0.938, *P* < 0.001) and CSA/BMI (OR = 0.065, *P* < 0.001) remained independently associated with lower odds of AECOPD, whereas conventional clinical variables were not independently significant. Bootstrap validation showed stable estimates. The combined model showed the highest discrimination, with an AUC of 0.806 (95% CI: 0.749–0.864), compared with %Vessel < 5, CSA/BMI, and FEV1%pred alone. At the optimal cutoff of 0.843, sensitivity was 62.3% and specificity was 89.7%. Decision curve and calibration analyses showed favorable clinical utility and calibration.

**Conclusion:**

Quantitative CT-derived CSA/BMI and %Vessel < 5 are independently associated with AECOPD and may provide complementary imaging information for characterizing patients with COPD.

## 1 Introduction

Chronic obstructive pulmonary disease (COPD) is characterized by persistent, largely irreversible airflow limitation and chronic respiratory symptoms. Acute exacerbation of COPD (AECOPD) is a key event associated with rapid symptom deterioration, accelerated lung function decline, impaired quality of life, and increased mortality (1). Large cohort studies have identified an “exacerbation-susceptible” phenotype, with prior exacerbation history being a strong predictor of future events (2). Cold exposure is also relevant: decreasing ambient temperature has been associated with increased COPD exacerbation incidence and higher AECOPD hospitalization risk (3, 4).

Although COPD diagnosis mainly relies on pulmonary function testing (5), airflow limitation alone cannot fully explain exacerbation susceptibility. Some patients with mild-to-moderate airflow limitation experience frequent exacerbations, indicating that pulmonary function indices may not adequately capture structural lung injury or systemic vulnerability (6). Chest CT can visualize emphysema and airway wall thickening and, through quantitative analysis, provide multidimensional biomarkers of lung parenchyma, pulmonary vasculature, and thoracic skeletal muscle. Such imaging features may complement COPD phenotyping, severity assessment, and prognostic evaluation (7).

COPD is frequently accompanied by systemic manifestations, particularly skeletal muscle dysfunction and wasting. CT-based studies have shown that thoracic skeletal muscle areas, including erector spinae and pectoralis muscles, may reflect COPD severity (8). Long-term follow-up further showed that reduced erector spinae cross-sectional area was strongly associated with mortality (9), suggesting that erector spinae parameters may reflect muscle reserve, nutritional status, and physiological vulnerability. Small pulmonary vascular injury is also involved in COPD progression. Wang et al. reported that the percentage of small pulmonary vessels with cross-sectional area <5 mm^2^ decreased with COPD severity, was lower in AECOPD than non-AECOPD patients, and correlated with FEV1%pred and FEV1/FVC (10).

Previous quantitative CT studies have mainly focused on general COPD populations, while imaging characteristics associated with AECOPD in cold-climate regions remain insufficiently understood. Therefore, this study assessed erector spinae and small pulmonary vessel parameters using chest quantitative CT, compared AECOPD and non-AECOPD patients, analyzed the associations of these CT parameters with pulmonary function, emphysema, and AECOPD status, and constructed a combined model to evaluate their joint discriminative value.

## 2 Materials and methods

### 2.1 Study population

This retrospective dual-center study initially screened 318 patients with COPD treated at Center 1 and Center 2 between January 2022 and October 2025. The inclusion criteria were as follows: (1) a diagnosis of COPD defined as a post-bronchodilator FEV1/FVC < 0.70; (2) availability of chest CT and pulmonary function testing during the same hospitalization; and (3) availability of clinical and imaging data required for quantitative analysis. AECOPD was defined according to the GOLD criteria as an acute worsening of dyspnea and/or cough and sputum over <14 days that was beyond normal day-to-day variation and required additional treatment. Patients meeting these criteria during the index hospitalization were classified into the AECOPD group. The non-AECOPD group comprised patients with established COPD who did not meet the criteria for AECOPD during the index hospitalization, including clinically stable patients and those hospitalized for other reasons without acute worsening of respiratory symptoms. For patients with AECOPD, chest CT was generally performed within 3 days after admission for the acute exacerbation. Pulmonary function testing was performed during the same hospitalization, generally within 2 weeks of CT and during the same clinical phase. The exclusion criteria were as follows: (1) obvious pulmonary infection, including tuberculosis or active inflammatory lesions (*n* = 23); and (2) lung cancer, a history of lung surgery, or other specified conditions that could affect quantitative CT assessment (*n* = 22). After application of the eligibility criteria, 273 patients were included in the final analysis, including 203 patients from Center 1 and 70 from Center 2. Among them, 58 patients were classified into the non-AECOPD group and 215 into the AECOPD group. The participant selection process is shown in Figure 1. Both centers are located in Harbin, Heilongjiang Province, northeastern China, where winters are long and cold, with an average minimum temperature of approximately −24 °C in January and a historical extreme minimum temperature of approximately −38 °C. This retrospective study was approved by the institutional ethics committees of the participating centers (approval numbers YJSKY2025-082 and KY2025-06) in May 2025 and September 2025, respectively. The requirement for written informed consent was waived because this was a retrospective, non-interventional study using anonymized clinical and imaging data.

**Figure 1:**
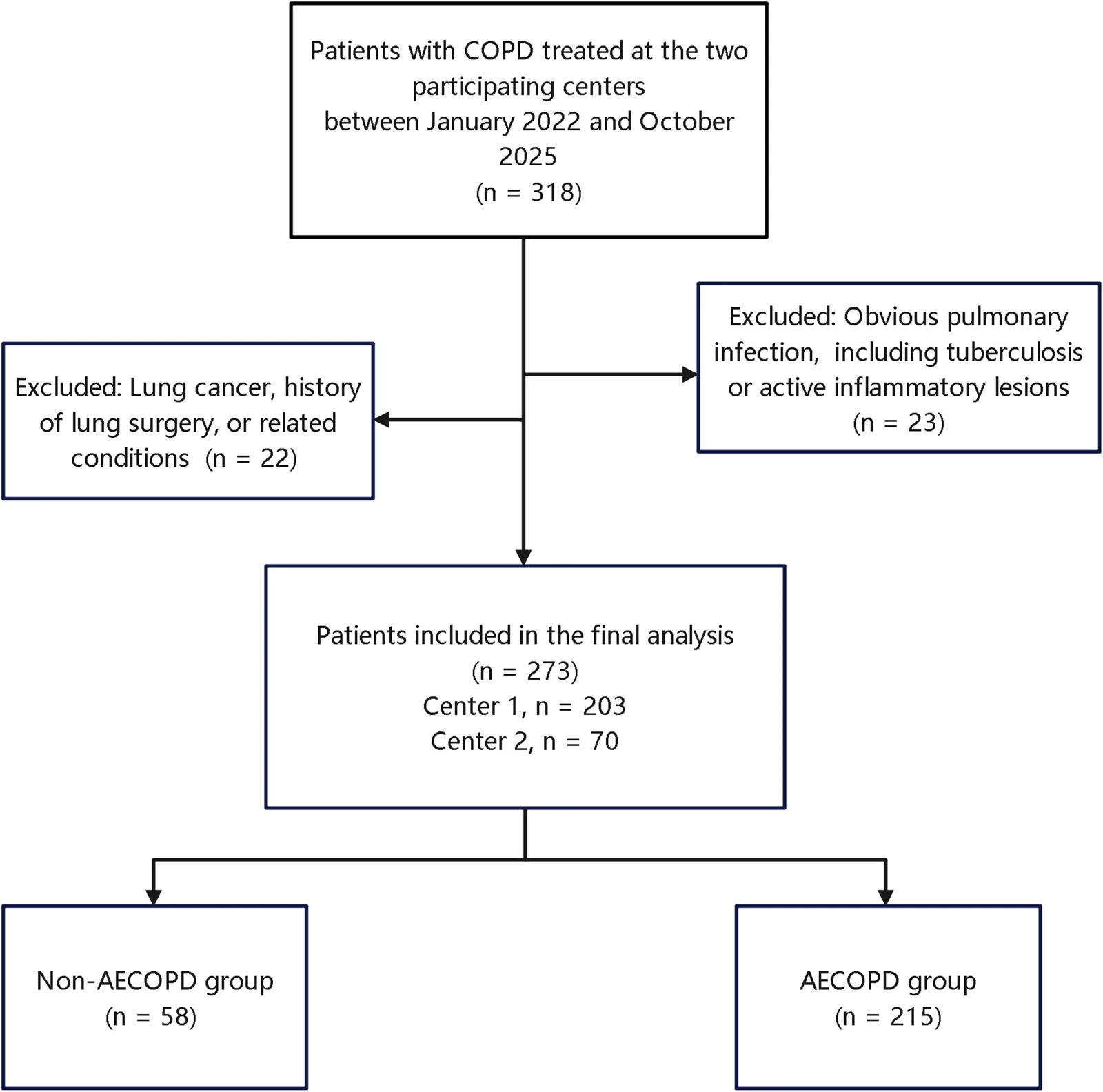
Flow diagram of participant selection. A total of 318 patients with COPD were initially screened at the two participating centers. After exclusion of 45 patients according to the exclusion criteria, 273 patients were included in the final analysis, including 58 patients in the non-AECOPD group and 215 patients in the AECOPD group.

### 2.2 CT acquisition

Chest CT was performed using a 128-slice Philips Brilliance iCT scanner or a 128-slice GE scanner. Patients were scanned from lung bases to apices at end-inspiration during breath-holding without contrast. Images used for quantitative analysis were reconstructed at 5-mm thickness and 5-mm intervals with 120 kV, 140 mA, a 512 × 512 matrix, and a bone algorithm. Because thin-section reconstructions were not consistently available in this retrospective cohort, 5-mm images were used for all quantitative analyses. Despite the use of different scanners, all images were processed using the same standardized ImageJ protocol, and study center was included as a covariate in the multivariable analysis.

### 2.3 Pulmonary function testing

Pulmonary function testing was performed using a Jaeger body plethysmograph. Parameters included vital capacity, total lung capacity, FEV1, FEV1/FVC, FEV1%pred, and DLCO. Tests were conducted seated, and the best of three repeated measurements was recorded.

### 2.4 Measurement of small pulmonary vessel parameters

Three CT slices were analyzed: 1 cm above the aortic arch, 1 cm below the carina, and 1 cm below the right inferior pulmonary vein (11). ImageJ 1.54p was used. Images were denoised by Gaussian blurring, lung fields were segmented from −500 to −1,024 HU, and binary images were generated at −720 HU (10). Small pulmonary vessels were extracted using “Analyze Particles”; vessels perpendicular or nearly perpendicular to the image plane were selected by circularity 0.9–1.0. Vessel areas <5 mm^2^ (Vessel < 5) and 5–10 mm^2^ (Vessel 5–10) were summed across three slices. Vascular density was calculated as vessel area divided by total lung area, expressed as %Vessel < 5 and %Vessel 5–10 (12–14). Two radiologists with 7 and 10 years of experience independently measured all parameters blinded to clinical data, and the mean value was used.

### 2.5 Quantitative analysis of erector spinae muscle parameters

Erector spinae muscles were measured using 3D Slicer 5.8.1. Left and right muscles were delineated at the inferior endplate of T12 and 3 cm above it, using −50 to 90 HU as the segmentation threshold. Parameters included CSA at T12, mean muscle attenuation, and local 3-cm muscle volume. Derived indices were CSA/height^2^, CSA/BMI, and CSA × CT (15). Lung segmentation was performed by thresholding, and areas below −950 HU were defined as emphysema to calculate LAA%. Measurements were independently performed by two radiologists, and mean values were used.

### 2.6 Statistical analysis

SPSS 27.0 was used. Normality was assessed by Shapiro–Wilk test. Normally distributed variables are presented as mean ± SD and compared by independent-samples *t*-test; non-normally distributed variables are presented as median (P25, P75) and compared by Mann–Whitney *U*-test. Categorical variables are presented as *n* (%) and compared by *χ*^2^-test. Spearman analysis assessed correlations among quantitative CT parameters and pulmonary function. Binary logistic regression evaluated associations with AECOPD, reported as ORs and 95% CIs. Variables with *P* < 0.20 in univariate analysis and clinically or methodologically relevant covariates were included in the multivariable model. Study center was retained to account for potential center-related differences. Because %Vessel < 5 values were small, they were multiplied by 100 for logistic regression; thus, the OR represents the change in the odds of AECOPD per 0.01% increase. ROC analysis evaluated discriminative performance, and pairwise comparisons of AUCs were performed using the DeLong test. Internal validation was performed using 1,000 bootstrap resamples. In each bootstrap sample, the complete multivariable model was refitted, and optimism was estimated by comparing model performance in the bootstrap sample with that in the original dataset. Optimism-corrected discrimination and calibration slope were subsequently calculated.

## 3 Results

### 3.1 Baseline characteristics

Baseline characteristics are shown in Table 1. Compared with the non-AECOPD group, the AECOPD group had significantly lower CSA, erector spinae volume, muscle attenuation, CSA/BMI, CSA × CT, and %Vessel < 5 (all *P* < 0.05). No significant differences were observed in general characteristics, smoking status, hospital source, or most pulmonary function parameters (all *P* > 0.05).

**Table 1:** Baseline characteristics of the non-AECOPD and AECOPD groups.

Variable	non-AECOPD group (*n* = 58)	AECOPD group (*n* = 215)	*P* value
General characteristics			
Age (years)	66.69 ± 6.64	68.30 ± 7.83	0.154
Sex (female/male), *n* (%)	23 (39.7)/35 (60.3)	103 (47.9)/112 (52.1)	0.263
Height (m)	1.65 (1.60, 1.70)	1.64 (1.58, 1.70)	0.268
Weight (kg)	59.07 ± 13.20	60.60 ± 13.40	0.439
BMI (kg/m^2^)	21.62 ± 4.04	22.61 ± 4.21	0.109
Smoking status (never/former/current), *n* (%)	28 (48.3)/4 (6.9)/26 (44.8)	113 (52.6)/31 (14.4)/71 (33.0)	0.137
Pack-years	6.25 (0.00, 40.00)	0.00 (0.00, 25.00)	0.162
Hospital (Center 1/Center 2), *n* (%)	44 (75.9)/14 (24.1)	159 (74.0)/56 (26.0)	0.768
Pulmonary function parameters			
FEV1/FVC (%)	55.59 (43.98, 62.52)	49.02 (41.50, 59.42)	0.052
FEV1%pred (%)	45.05 (32.93, 59.23)	38.30 (29.60, 51.20)	0.108
FVC (%)	61.15 (47.60, 76.13)	58.40 (48.50, 71.20)	0.434
FEV1 (%)	41.20 (30.15, 55.20)	33.80 (26.50, 50.30)	0.104
TLC (%)^a^	70.95 (61.00, 78.93)	70.40 (60.13, 82.15)	0.617
RV (%)^a^	100.10 (84.00, 116.58)	100.50 (83.53, 132.75)	0.482
DLco (%)^b^	43.70 (33.75, 59.20)	43.00 (28.70, 56.95)	0.434
DLco/VA^c^	0.89 (0.63, 1.30)	0.80 (0.55, 1.12)	0.154
DLco/VA (%)^c^	64.10 (48.60, 95.00)	60.10 (39.40, 79.45)	0.209
Quantitative CT parameters			
LAA (%)	12.67 (6.63, 20.30)	12.55 (7.73, 18.90)	0.855
CSA (cm^2^)	33.75 (30.50, 39.52)	32.57 (27.88, 38.18)	0.037
Volume (cm^3^)	108.25 ± 26.05	100.39 ± 23.09	0.026
CT attenuation (HU)	45.17 (38.98, 51.04)	41.23 (34.76, 47.11)	0.008
%Vessel < 5, per 0.01% increase	38.95 (34.00, 45.99)	32.15 (24.00, 40.26)	<0.001
%Vessel 5–10,per 0.01% increase	0.299 (0.242, 0.370)	0.290 (0.222, 0.352)	0.331
CSA/height^2^	12.90 (11.66, 14.02)	12.33 (10.64, 14.15)	0.069
CSA/BMI	1.68 ± 0.29	1.48 ± 0.29	<0.001
CSA × CT	1,522.76 (1,211.32, 1,946.25)	1,353.28 (977.54, 1,668.65)	0.002

AECOPD, acute exacerbation of chronic obstructive pulmonary disease, BMI, body mass index, FEV1, forced expiratory volume in 1 s, FVC, forced vital capacity, TLC, total lung capacity, RV, residual volume, DLco, diffusing capacity of the lung for carbon monoxide, VA, alveolar volume, CT, computed tomography, LAA, low attenuation area, CSA, cross-sectional area, HU, Hounsfield unit. %Vessel < 5 mm^2^ and %Vessel 5–10 mm^2^ indicate the percentage of total lung area occupied by pulmonary vessels with cross-sectional areas of <5 mm^2^ and 5–10 mm^2^, respectively.

Data are presented as mean ± standard deviation, median (P25, P75), or *n* (%), as appropriate. Normally distributed variables were compared using the independent-samples *t*-test, non-normally distributed variables using the Mann–Whitney *U*-test, and categorical variables using the chi-square test.

a Valid cases for TLC and RV: 54 in the non-AECOPD group and 184 in the AECOPD group.

b Valid cases for DLCO: 48 in the non-AECOPD group and 145 in the AECOPD group.

c Valid cases for DLCO/VA and DLCO/VA%: 51 in the non-AECOPD group and 162 in the AECOPD group.

### 3.2 Univariate logistic regression

Univariate analysis showed that CSA, volume, muscle attenuation, CSA/height^2^, CSA/BMI, CSA × CT, and %Vessel < 5 (per 0.01% increase) were significantly associated with AECOPD (all *P* < 0.05), with ORs <1, indicating lower odds of AECOPD. Age, FEV1%pred, and pack-years had *P* < 0.20 and were included in multivariate analysis; center effect was included for adjustment.

### 3.3 Correlation and multivariate logistic regression

Spearman analysis showed positive correlations among erector spinae parameters. %Vessel < 5 correlated positively with FEV1/FVC (*r* = 0.354, *P* < 0.001) and FEV1%pred (*r* = 0.226, *P* < 0.001), and negatively with LAA% (*r* = −0.410, *P* < 0.001). CSA/BMI was not significantly correlated with %Vessel < 5 (*r* = −0.097, *P* = 0.111) or FEV1%pred (*r* = 0.012, *P* = 0.848), suggesting limited overlap between skeletal muscle reserve and pulmonary vascular alteration. As shown in Table 2, multivariate analysis showed that %Vessel < 5 (OR = 0.938, 95% CI: 0.912–0.964, *P* < 0.001) and CSA/BMI (OR = 0.065, 95% CI: 0.019–0.216, *P* < 0.001) were independently associated with lower odds of AECOPD, whereas conventional clinical variables were not independently significant. Internal validation using 1,000 bootstrap resamples yielded an optimism-corrected AUC of 0.784, compared with an apparent AUC of 0.806, and an optimism-corrected calibration slope of 0.881, indicating modest model optimism.

**Table 2:** Logistic regression and bootstrap validation for AECOPD.

Variables	Univariate OR (95% CI)	*P* value	Multivariable OR (95% CI)	*P* value	Bootstrap 95% CI*
Clinical Characteristics					
Age, years	1.028 (0.990–1.068)	0.154	1.012 (0.968–1.059)	0.590	0.968–1.059
Hospital center	0.903 (0.460–1.773)	0.768	1.078 (0.506–2.297)	0.845	0.470–2.275
Smoking history, pack-years	0.991 (0.981–1.002)	0.102	0.999 (0.987–1.011)	0.873	0.988–1.013
FEV1%pred, %	0.988 (0.974–1.002)	0.089	0.993 (0.977–1.009)	0.398	0.976–1.011
Quantitative CT parameters					
%Vessel < 5, per 0.01% increase	0.946 (0.924–0.970)	<0.001	0.938 (0.912–0.964)	<0.001	0.900–0.965
CSA/BMI, cm^2^/(kg/m^2^)	0.103 (0.037–0.291)	<0.001	0.065 (0.019–0.216)	<0.001	0.016–0.179

AECOPD, acute exacerbation of chronic obstructive pulmonary disease, OR, odds ratio, CI, confidence interval, FEV1, forced expiratory volume in 1 s, FEV1%pred, percentage of predicted FEV1, CT, computed tomography, CSA, cross-sectional area, BMI, body mass index. %Vessel < 5 indicates the percentage of total lung area occupied by pulmonary vessels with cross-sectional areas of < 5 mm^2^. CSA/BMI indicates the erector spinae muscle cross-sectional area divided by body mass index. Hospital center was coded as Center 2 vs. Center 1.

Bootstrap validation was performed using 1,000 resamples. Bootstrap 95% CIs are presented for the multivariable model. For %Vessel < 5, the original value was multiplied by 100 before logistic regression analysis; therefore, the OR represents the change in AECOPD risk for each 0.01% increase in %Vessel < 5.

### 3.4 Model discrimination and clinical utility

ROC analysis showed that the joint model had the best discrimination, with an AUC of 0.806 (95% CI: 0.749–0.864), compared with %Vessel < 5 (AUC = 0.702), CSA/BMI (AUC = 0.681), and FEV1%pred (AUC = 0.569) (Figure 2). DeLong tests showed that the AUC of the combined model was significantly higher than those of %Vessel < 5 (*P* = 0.002), CSA/BMI (*P* < 0.001), and FEV1%pred (*P* < 0.001). The optimal cutoff was 0.843, with a Youden index of 0.520, sensitivity of 62.3%, and specificity of 89.7%. A nomogram integrating hospital source, age, pack-years, FEV1%pred, %Vessel < 5, and CSA/BMI was constructed (Figure 3). Lower %Vessel < 5, CSA/BMI, and FEV1%pred corresponded to a higher estimated probability of AECOPD. Decision curve analysis showed that the combined model provided greater net benefit than the single-indicator models across threshold probabilities of 0.43–0.76 (Figure 4). This range represents hypothetical decision thresholds at which the expected benefit of model-guided additional assessment may outweigh the consequences of unnecessary assessment; however, it should not be interpreted as a validated clinical intervention threshold. Calibration analysis showed good agreement between the estimated probabilities and observed frequencies of AECOPD; the Hosmer–Lemeshow test supported good fit (*χ*^2^ = 11.985, df = 8, *P* = 0.152) (Figure 5). Bootstrap internal validation yielded an optimism-corrected AUC of 0.784 and an optimism-corrected calibration slope of 0.881, indicating modest model optimism.

**Figure 2:**
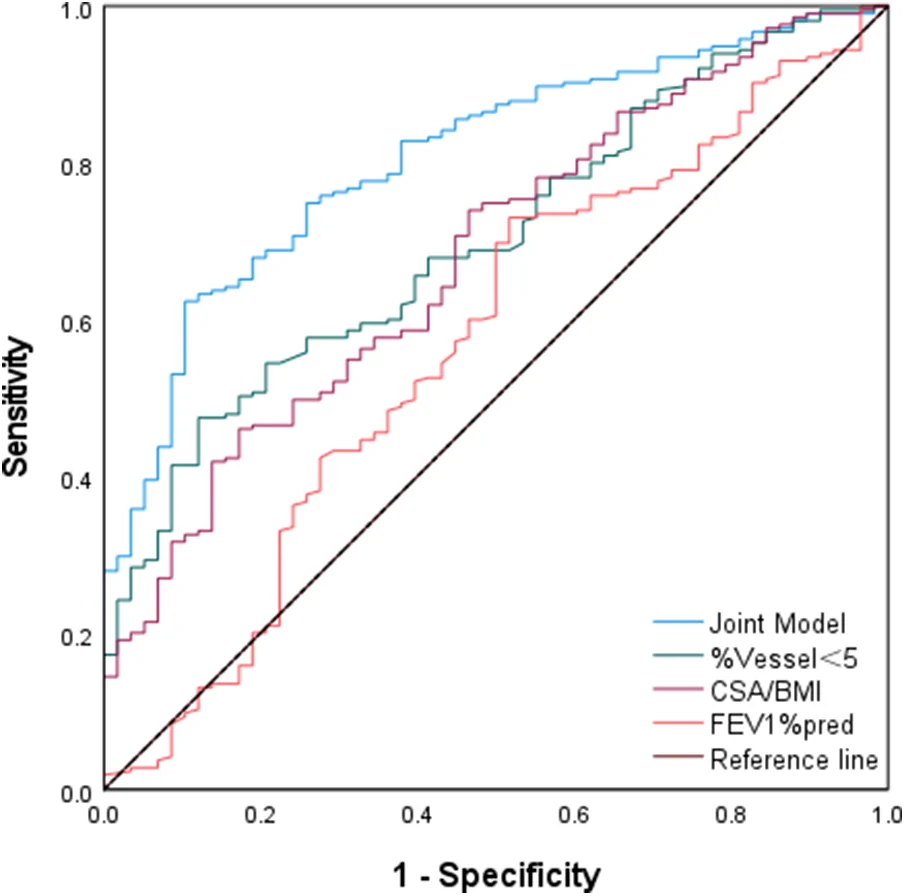
ROC curves of individual indicators and the joint model. For the protective factors %Vessel < 5 and CSA/BMI, ROC curves were generated using their inverse values to consistently represent their ability to predict increased AECOPD risk and to ensure that all curves reflected the same outcome direction.

**Figure 3:**
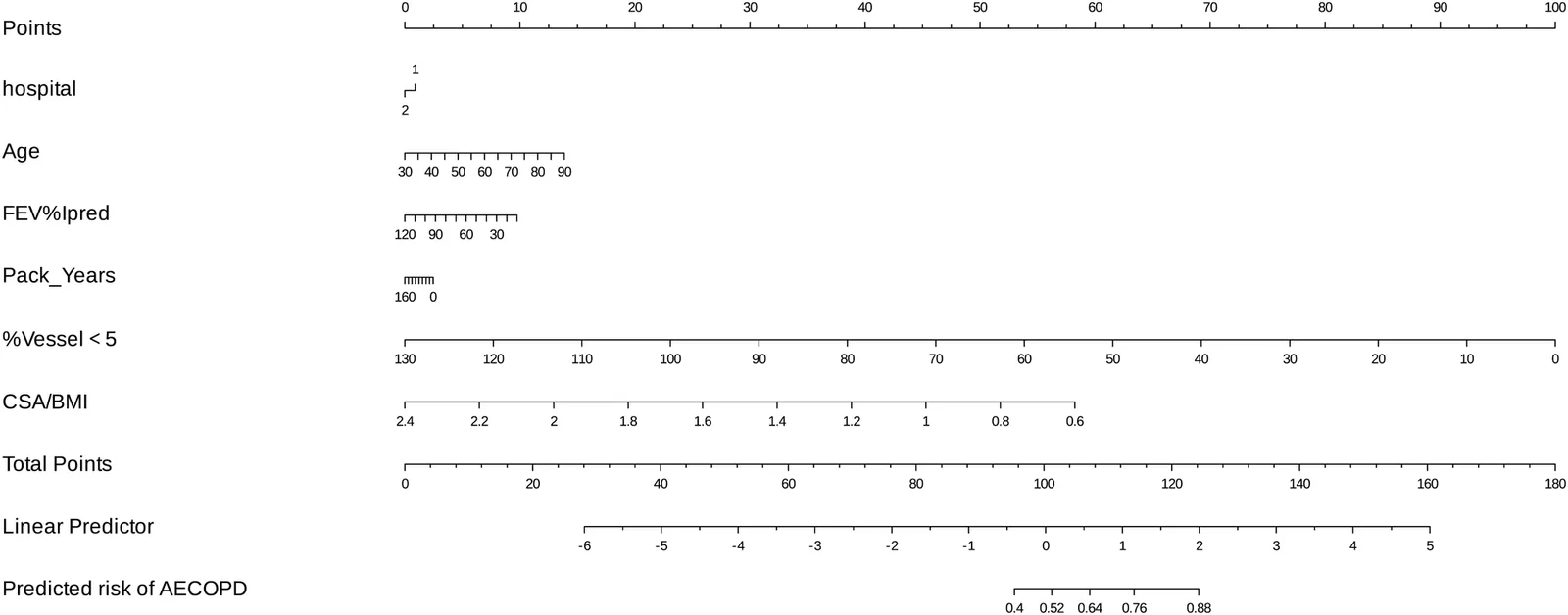
Nomogram of the combined AECOPD model. The nomogram was constructed using hospital source, age, pack-years, FEV1%pred, %Vessel < 5, and CSA/BMI. For the hospital variable, 1 indicates Center 1, and 2 indicates Center 2.

**Figure 4:**
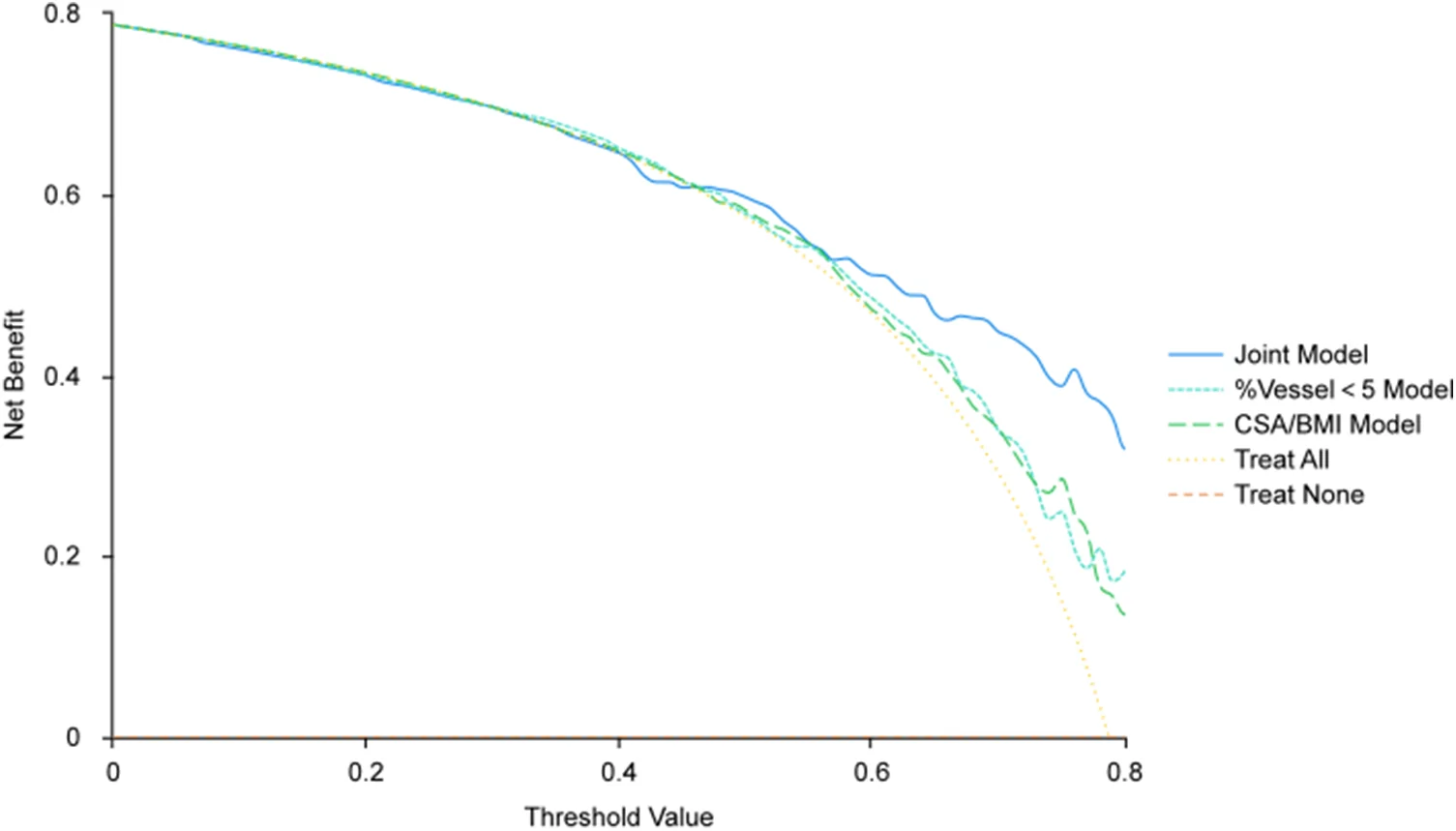
Decision curve analysis of the combined AECOPD model. The combined model, %Vessel < 5-only model, and CSA/BMI-only model were compared with the treat-all and treat-none strategies to evaluate clinical net benefit across different threshold probabilities.

**Figure 5:**
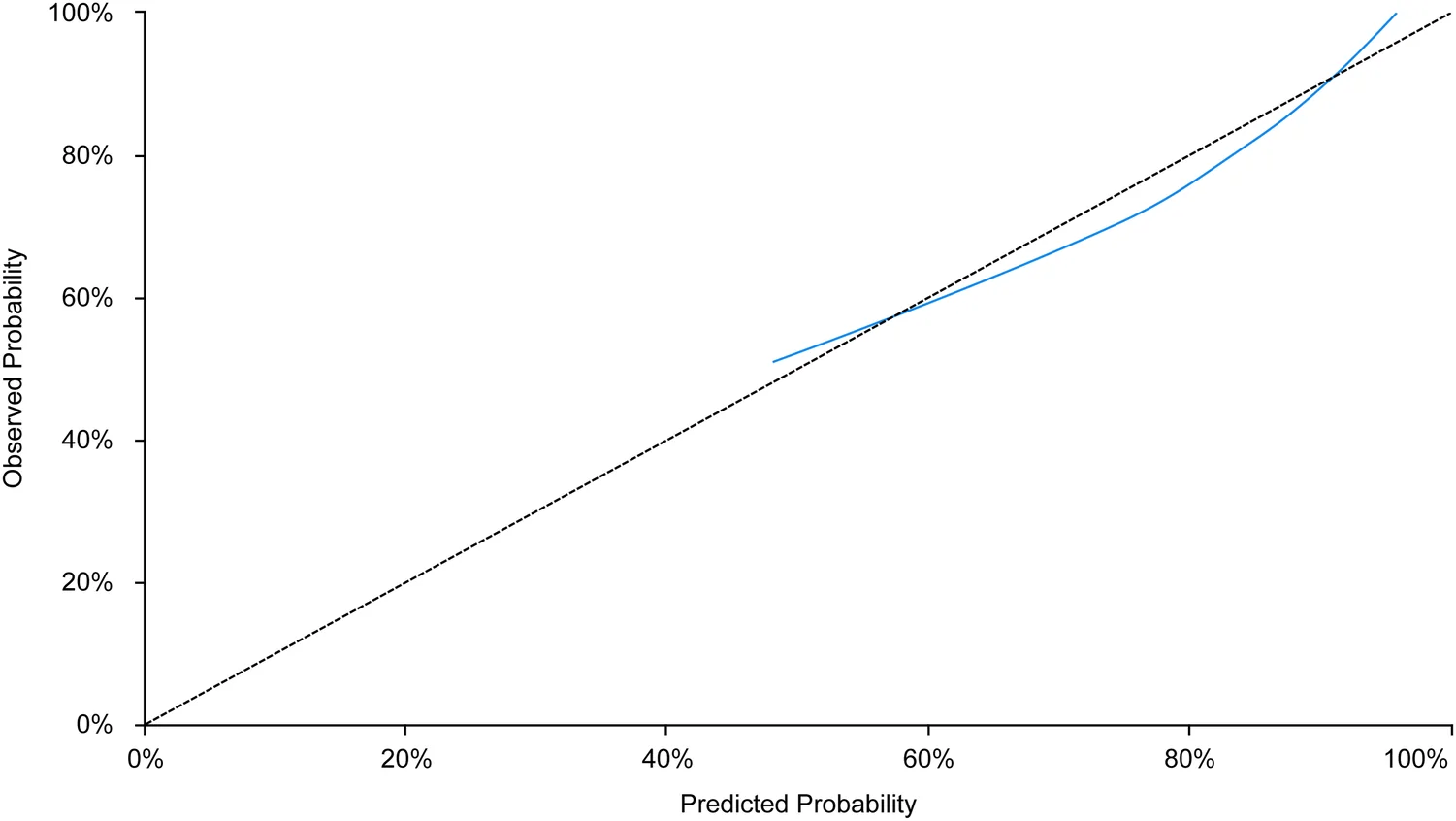
Calibration curve of the combined AECOPD model. The blue solid line represents the combined prediction model developed in this study, and the black 45-degree dashed line represents ideal calibration.

## 4 Discussion

In this dual-center cold-region cohort, quantitative CT-derived %Vessel < 5 and CSA/BMI were independently associated with AECOPD after adjustment for age, smoking history, pulmonary function, and center effect. Conventional indicators, including FEV1%pred, did not retain independent significance after inclusion of these imaging markers. These findings suggest that joint assessment of pulmonary microvascular preservation and skeletal muscle reserve may capture COPD heterogeneity that is not fully reflected by airflow limitation alone. Since multiple factors contribute to AECOPD susceptibility, a single indicator is unlikely to fully characterize AECOPD mechanisms (16).

An important contextual feature of this study is that both participating centers are located in Harbin, a cold-climate region of northern China. The regional temperature data provide an objective climatic description of the study setting but should not be interpreted as individual-level exposure measurements. The low winter temperatures in Harbin provide an objective climatic basis for this regional classification. Previous studies on CT-derived small pulmonary vessels and skeletal muscle have rarely addressed cold-climate exposure, although low temperature is associated with increased COPD exacerbation and hospitalization risk (3, 4). Cold air may aggravate airway inflammation and infection susceptibility, while inhalation of cold, dry air can induce airway cooling and dehydration, leading to bronchial hyperresponsiveness and reflex bronchoconstriction. Cold exposure may also increase pulmonary vascular stress and reduce physical activity, potentially amplifying the effects of pulmonary microvascular injury and limited skeletal muscle reserve. Together with infection, these mechanisms may increase susceptibility to AECOPD (17). These climatic characteristics provide relevant context for interpreting the observed associations in this cold-region cohort.

Reduced %Vessel < 5 was closely associated with greater odds of AECOPD. Pulmonary vascular abnormalities have gained attention in COPD. Beyond airway obstruction and emphysema, endothelial dysfunction, hypoxic vasoconstriction, and vascular remodeling may reduce the distal pulmonary vascular bed, appearing as vascular “pruning” on imaging (18, 19). Loss of small vessels may aggravate ventilation/perfusion mismatch, impair pulmonary circulatory reserve, and relate to lung function decline, emphysema progression, and increased right ventricular load (20). When exposed to infection, low temperature, or air pollution, patients with pre-existing vascular impairment may be more vulnerable to ventilation, perfusion, and oxygenation imbalance. Thus, %Vessel < 5 may be a useful imaging marker for characterizing patients with AECOPD.

This study also supports the systemic nature of COPD. COPD is frequently accompanied by systemic inflammation, nutritional depletion, skeletal muscle dysfunction, and extrapulmonary comorbidities (21). Higher CSA/BMI was independently associated with lower odds of AECOPD, suggesting that preserved skeletal muscle reserve may be related to a less vulnerable systemic phenotype. Sarcopenia is common in COPD and may reflect chronic inflammation, malnutrition, and inactivity; it may also impair airway clearance by weakening respiratory and core muscles (22, 23). BMI cannot distinguish fat from muscle, whereas chest CT-based skeletal muscle quantification directly evaluates functional muscle reserve. CT-measured erector spinae or pectoralis muscle area has been associated with COPD severity and prognosis (24), supporting its value as a complementary imaging marker.

Although FEV1/FVC and FEV1%pred were numerically lower in the AECOPD group, the differences did not reach statistical significance, while LAA% was similar between groups. This may partly reflect the substantial overlap in COPD severity between the groups, as the non-AECOPD group also consisted of patients with established COPD rather than healthy controls or patients selected for mild disease. The relatively high proportion of never-smokers, particularly in the AECOPD group, also contributed to the low median pack-year values, while neither smoking status nor pack-years differed significantly between groups. The loss of independent significance for FEV1%pred after inclusion of QCT markers does not diminish the diagnostic role of pulmonary function testing; rather, it indicates that ventilatory function alone may be insufficient for characterization of AECOPD. The nomogram transformed %Vessel < 5 and CSA/BMI into individualized estimated probabilities of AECOPD, improving clinical interpretability. Decision curve analysis further suggested clinical net benefit within relevant threshold ranges, supporting the potential value of the model as an adjunct to clinical characterization of AECOPD. However, it should be combined with dynamic clinical information, including symptoms, infection markers, and environmental exposure (25, 26).

These findings may also be relevant to COPD populations in other regions characterized by prolonged low winter temperatures, where similar cold-related respiratory and systemic stressors may occur. However, regional differences in temperature patterns, humidity, air pollution, population characteristics, and healthcare settings may influence generalizability, warranting further multicenter validation in other cold-climate regions.

Several limitations should be acknowledged. First, because chest CT was obtained during the same hospitalization in which exacerbation status was determined, generally within 3 days after admission for AECOPD, temporal causality cannot be established. Acute inflammation, fluid shifts, treatment effects, or short-term inactivity during exacerbation may have influenced pulmonary vascular and skeletal muscle measurements; therefore, reverse causation cannot be excluded. Second, this retrospective dual-center study lacked external validation, and although bootstrap validation suggested model stability, its generalizability requires further evaluation. Third, only single-time-point imaging data were analyzed, and longitudinal changes in %Vessel < 5 and skeletal muscle parameters were n,ot assessed. Small pulmonary vessel measurements were based on 5-mm reconstructed images, which may be more susceptible to partial-volume effects, while thin-section and adequately powered center-stratified sensitivity analyses were not feasible in this retrospective cohort. Finally, although regional temperature data were used to characterize the cold-climate setting, individual-level meteorological exposure data and comparison cohorts from other climatic regions were unavailable; therefore, the specific contribution of cold exposure to the observed associations could not be determined. Future multicenter prospective studies incorporating meteorological monitoring and longitudinal imaging are needed.

## 5 Conclusion

AECOPD was associated with alterations in both pulmonary small-vessel and skeletal muscle parameters, with lower %Vessel < 5 and CSA/BMI remaining independently associated with AECOPD after multivariable adjustment. These findings suggest that AECOPD may involve both local pulmonary injury and systemic depletion. The combined assessment of %Vessel < 5 and CSA/BMI showed good discrimination between the study groups and may provide complementary imaging information alongside conventional clinical measures. These findings support the potential value of quantitative CT for multidimensional characterization of COPD; however, prospective longitudinal studies are needed to determine whether these markers can predict future exacerbations.

## Data Availability

The raw data supporting the conclusions of this article will be made available by the authors, without undue reservation.
